# Modified Z‐resolution protocol for digital breast tomosynthesis

**DOI:** 10.1002/mp.70625

**Published:** 2026-08-25

**Authors:** Priyash Singh, Nyasha G. Maforo, Bruno Barufaldi, Andrew D. A. Maidment, Raymond J. Acciavatti

**Affiliations:** ^1^ Department of Radiology University of Pennsylvania Philadelphia Pennsylvania USA

**Keywords:** artifact spread function, digital breast tomosynthesis, point spread function

## Abstract

**Background:**

The EUREF protocol measures the z‐resolution of a digital breast tomosynthesis (DBT) system via a ball bearing's (BB) point‐spread function (PSF) and is quantified as the full‐width at half‐maximum (FWHM) of the associated artifact spread function (ASF). PSF is largely governed by the apparent angle (α), defined as the radian angle subtended by the BB across the X‐ray sources, which in turn depends on the BB's location and acquisition geometry. Under fixed DBT geometry and reconstruction conditions, the FWHM is therefore expected to follow a predictable spatial trend. However, we found that the EUREF protocol does not consistently reproduce this behavior; it fails to capture the gradual rise in FWHM for anteriorly displaced BBs expected from a narrowing α (with which FWHM is inversely correlated) and exhibits a systematic bias to the stochastic noise in the reconstruction image.

**Purpose:**

The purpose of this work was to develop a z‐resolution metric that more rigorously reflects the underlying local PSF's α without incurring a sensitivity to stochastic noise. We propose a protocol that uses multiplanar reconstruction to track the oblique BB signal in its natural spatial scale, uses means instead of maxima to generate the ASF, and employs a functional fit to estimate the FWHM more robustly—hereafter referred to as the Modified Protocol.

**Methods:**

The protocol was evaluated using analytically simulated and experimentally acquired DBT reconstructions of BBs displaced anteriorly and superiorly and compared against the EUREF protocol. The percent change in FWHM due to stochastic noise was used to quantify systematic noise susceptibility. The strength of the anti‐correlation between FWHM and apparent angle α was used to assess each protocol's agreement with the PSF geometry.

**Results:**

By demonstrating a consistent linear anti‐correlation between FWHM and α for both superior and anterior BB displacements, the Modified protocol faithfully captures the spatial variation in PSF. The Modified protocol also substantially reduces the noise‐induced FWHM shift—by 98% in simulations and 84% in physical experiments—relative to the EUREF protocol.

**Conclusions:**

The EUREF protocol exhibits systematic errors which can limit its reliability in characterizing the z‐resolution. Our Modified protocol mitigates these shortcomings and restores concordance between measured z‐resolution and the underlying local PSF geometry (α), while also improving precision by reducing the susceptibility to stochastic noise. The Modified protocol thus offers an alternative to the existing EUREF framework, providing a z‐resolution metric that serves as a more reliable surrogate for the PSF while remaining more robust to image noise.

**Abbreviations**
ASF: artifact spread function
BB: ball bearing
Bgd: background
CW: chest wall
DBT: digital breast tomosynthesis
FWHM: full width at half maximum
mAs: milliampere‐seconds
MPR: multi‐planar reconstruction
MTF: modulation transfer function
NGT: next‐generation tomosynthesis
NPS: noise power spectrum
PSF: point spread function
R^2^
: coefficient of determination
ROI: region of interest
SBP: simple back‐projection
SNR: signal‐to‐noise ratio
uASF: unnormalized artifact spread function
uASF_fit_
: parametric fit of the uASF

## 1 INTRODUCTION

Digital breast tomosynthesis (DBT) represents a significant advancement in breast cancer screening, offering notable improvements over traditional 2D digital mammography. Many of its benefits, such as higher cancer detection rates, stem from its ability to provide enhanced z‐resolution that resolves tissue overlap. As DBT gains wider adoption, it becomes imperative to quantify its depth resolution reliably to ensure patients continue to receive its benefits.

The European reference organization for quality assured breast screening and diagnostic services (EUREF) prescribes a protocol to quantify DBT's z‐resolution using the reconstruction of a spherical ball bearing (BB).^1^ In the focal slice, a region of interest (ROI) is drawn around the BB and another is placed in the background (Bgd) nearby. After ensuring the BB‐ROI is large enough to encompass the laterally shifting out‐of‐plane artifacts between adjacent slices, the planar ROIs are then extended vertically to all slices. To quantify signal spread, the protocol defines the artifact spread function (ASF) as the normalized difference between the BB signal and Bgd profiles along the vertical axis (z). This unnormalized ASF is first obtained by computing $\mathrm{uASF}\left(z\right)\overset{\text{def}}{=}I_{\mathrm{BB}}^{\mathrm{RO}I_{\max}}\left(z\right)-I_{\mathrm{Bgd}}^{\mathrm{RO}I_{\mathrm{avg}}}\left(z\right)$, which subtracts the Bgd‐ROI mean from the BB‐ROI maximum on each slice to suppress underlying trends. Normalizing this difference to its peak value in the focal slice (z_0_) yields the desired $\mathrm{ASF}\left(z\right)\overset{\text{def}}{=}\mathrm{uASF}\left(z\right)/\mathrm{uASF}\left(z_{0}\right)$. Typically, as the z‐separation from the focal slice increases, the ASF(z) decreases. The z‐resolution is determined from the full‐width at half‐maximum (FWHM) defined as the separation between z‐slices where ASF(z) has fallen to 0.5 in magnitude. The EUREF protocol may be mathematically formalized as,

(1) $$\mathrm{ASF}\left(z\right)\overset{\text{def}}{=}\frac{I_{\mathrm{BB}}^{\mathrm{RO}I_{\max}}\left(z\right)-I_{\mathrm{Bgd}}^{\mathrm{RO}I_{\mathrm{avg}}}\left(z\right)}{I_{\mathrm{BB}}^{\mathrm{RO}I_{\max}}\left(z_{0}\right)-I_{\mathrm{Bgd}}^{\mathrm{RO}I_{\mathrm{avg}}}\left(z_{0}\right)}=\frac{\mathrm{uASF}\left(z\right)}{\mathrm{uASF}\left(z_{0}\right)}$$

(2) $$\mathrm{FWH}M_{\mathrm{EUREF}}\overset{\text{def}}{=}z_{1}-z_{2}\;s.t.\;\mathrm{ASF}\left(z_{1,2}\right)=0.5$$

**FIGURE 1:**
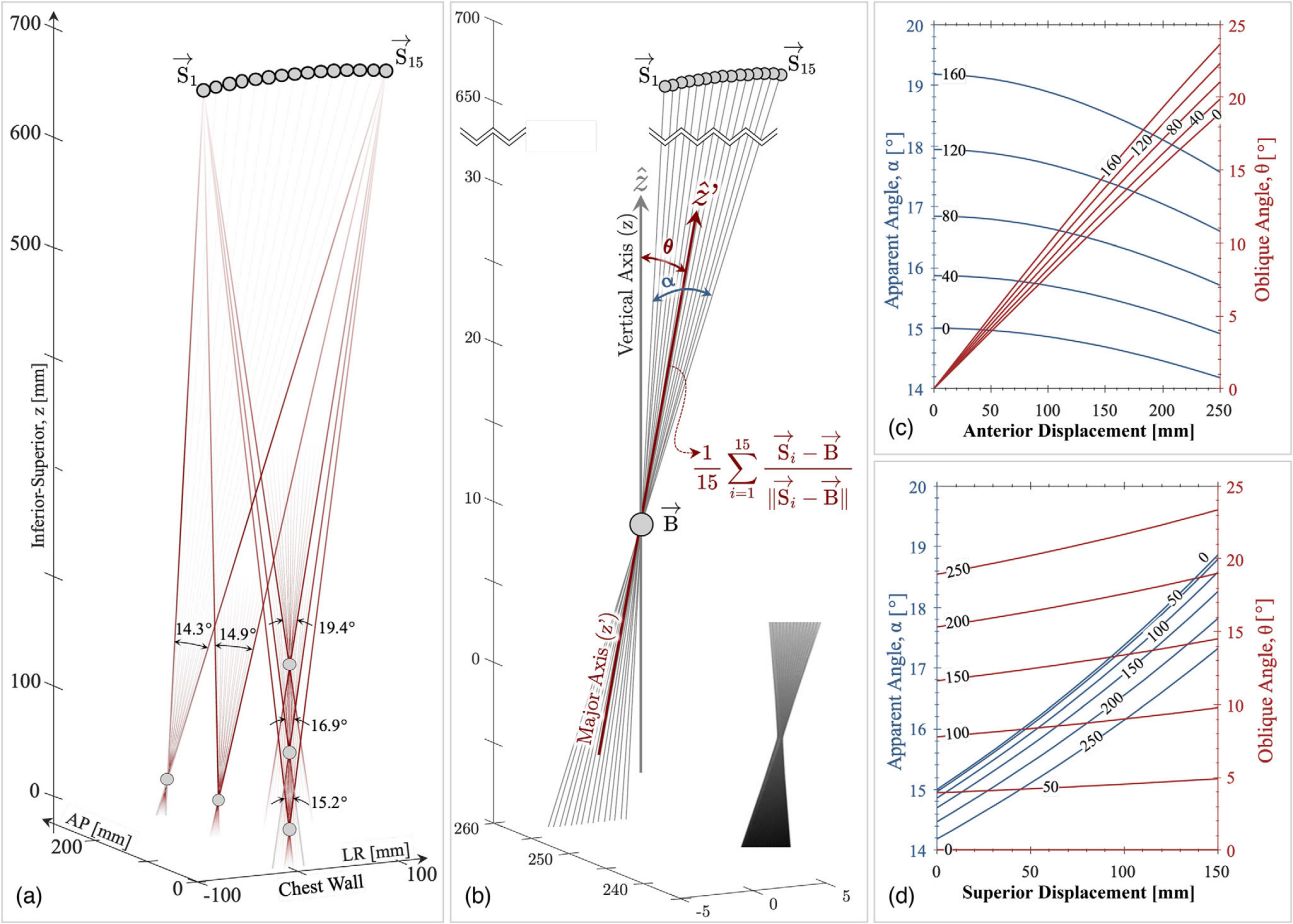
Geometric view of the influence of BB position apparent and oblique angles for a DBT system. (a) Schematic illustrating apparent angle's variation with BB displacements along the midplane. Superior displacements increase the apparent angle, while anterior displacements reduce it. AP: Anterior‐Posterior, LR: Left‐Right (b) Magnified view of one such BB showing the associated apparent, oblique angles and the orientations of PSF's major axis, computed as the average of the unit vectors from the BB to each of the 15 X‐ray sources. 3D rendering of the PSF is also depicted on the bottom right (c–d) Variation of apparent angle (left y‐axis in blue) and oblique angle (right y‐axis in red) plotted against displacements along the midplane. In (c), the angles are evaluated as a function of anterior displacement for multiple heights above the breast support (0–160 mm) and in (d), they are evaluated as a function of superior displacement at multiple anterior offsets from the chest wall (0–250 mm).

The EUREF protocol essentially attempts to quantify the 3D Point‐Spread Function (PSF) describing the BB's reconstructed signal. In DBT the PSF is tilted, with its major axis oriented toward the X‐ray sources (Figure 1b). The PSF becomes increasingly oblique away from the chest wall (CW), causing the BB signal to defocus laterally between z‐slices. This is why extracting a strictly vertical profile at fixed (x,y) through the BB is not representative of the intensities along the tilted major axis. Such a vertical path will cut across the BB's 3D PSF instead of being contained within its double cone. The EUREF protocol addresses this challenge by defining the ASF(z) using maximum of the BB‐ROI, acting as a surrogate measure of signal along the PSF's major axis. By doing so, the protocol has shown some success in capturing the variation of z‐resolution within the reconstruction volume as would be expected from changes in the PSF's geometrical shape.

To illustrate these spatial trends in z‐resolution, we examine the effect of BB position within the reconstruction volume. Here, we introduce a geometric descriptor of the local PSF through the apparent angle (α), defined as the angle subtended by the BB across the X‐ray source trajectory (Figure 1b). It reflects the effective angular range of the DBT acquisition as perceived by the BB. As the BB is displaced superiorly, this apparent angle increases, improving localization of signal along the PSF's major axis and thereby improving z‐resolution (Figure 1a,d). This reduction in FWHM of the ASF(z) from increasing apparent angle has indeed been supported by several studies employing the EUREF protocol. Kuramoto et al. found the FWHM to decrease (implying improved z‐resolution) by ∼15% over an elevation of 150 mm on a SONIALVISION G4 unit.^2^ Nishioka et al. found an 8.5% decrease over a 40 mm increase in height.^3^ Rodríguez‐Ruiz et al. found a slight 5%–20% decrease over a height of 40 mm.^4^ Similarly, the EUREF protocol has also been found to correctly capture FWHM reduction resulting from an increased angular range in DBT acquisition geometry itself, which would also naturally increase α. Zhou et al. reported a 50% decrease in FWHM when the angular range was increased from 15° to 30° which is in sound agreement with results from Hu et al., Sechopoulos and Ghetti, Dalmonte et al. and Nosratieh et al. as well.^5^, ^6^, ^7^, ^8^, ^9^

Having established that the EUREF protocol captures expected z‐resolution variations in the superior direction or those associated with the angular range of the acquisition geometry itself, we extend this examination to the anterior direction. The apparent angle decreases with anterior displacement of the BB (Figure 1c) so we should therefore expect poorer z‐resolution (wider FWHM) here. This trend, however, has not been observed consistently across experimental studies employing the EUREF protocol. Rodríguez‐Ruiz et al. do present data suggesting a weak increase in FWHM for anteriorly displaced BBs, but stop short of recognizing it as a significant trend and instead remark on the poor reproducibility of ASF(z) away from the CW.^4^ Maki et al. report a 5% decrease in FWHM over 100 mm of anterior displacement, contradicting the expected inverse relationship between α and FWHM.^10^ Nishioka et al. found an 8% reduction in FWHM with 225 mm of anterior displacement, again inconsistent with the expected relationship.^3^ To our knowledge, no study has provided consistent experimental evidence of an increasing FWHM with anterior displacement as would be expected from a narrowing apparent angle in this direction.

In this work, we aim to reconcile this inconsistency. Through theoretical modeling, we demonstrate that the EUREF protocol exhibits two opposing systematic errors: it underestimates FWHM for anteriorly displaced BBs due to (i) PSF obliquity and (ii) Max‐based ASF sampling strategy yet overestimates FWHM through (iii) Its sensitivity to stochastic noise in the reconstruction and (iv) FWHM computation methodology. Taken together, these observations introduce variable biases that prevent the EUREF protocol from accurately reflecting the geometric relationship between the local PSF's apparent angle and z‐resolution, while also introducing significant uncertainty in FWHM measurement. To overcome these shortcomings, we propose a new protocol designed to mitigate such systematic biases, hereafter referred to as the Modified protocol. We evaluate its performance using both simulations and reconstructions of a physical phantom with embedded BBs at various positions, demonstrating its FWHM trends to be more consistently aligned with the expected behavior of the PSF's apparent angle—namely, that anterior and superior BB displacements lead to degradations and improvements in z‐resolution, respectively.

## 2 REVIEW OF EUREF PROTOCOL

### 2.1 Systematic underestimation of FWHM

The EUREF protocol prescribes using the maxima of the BB‐ROIs as a putative surrogate for the signal along the 3D PSF's tilted major axis. Relying on the maxima, however, creates a discrepancy where the uASF extracted at each slice is not the profile along the intended major axis (z′) but along an intermediate axis (z″), that lies in between the major (z′) and vertical (z) axes. Expressing this profile against the abscissa of the slice location (z) rather than its own intrinsic axis (z″), further compounds the discrepancy, producing an artificially narrowed uASF profile. This leads to an underestimation in FWHM.

**FIGURE 2:**
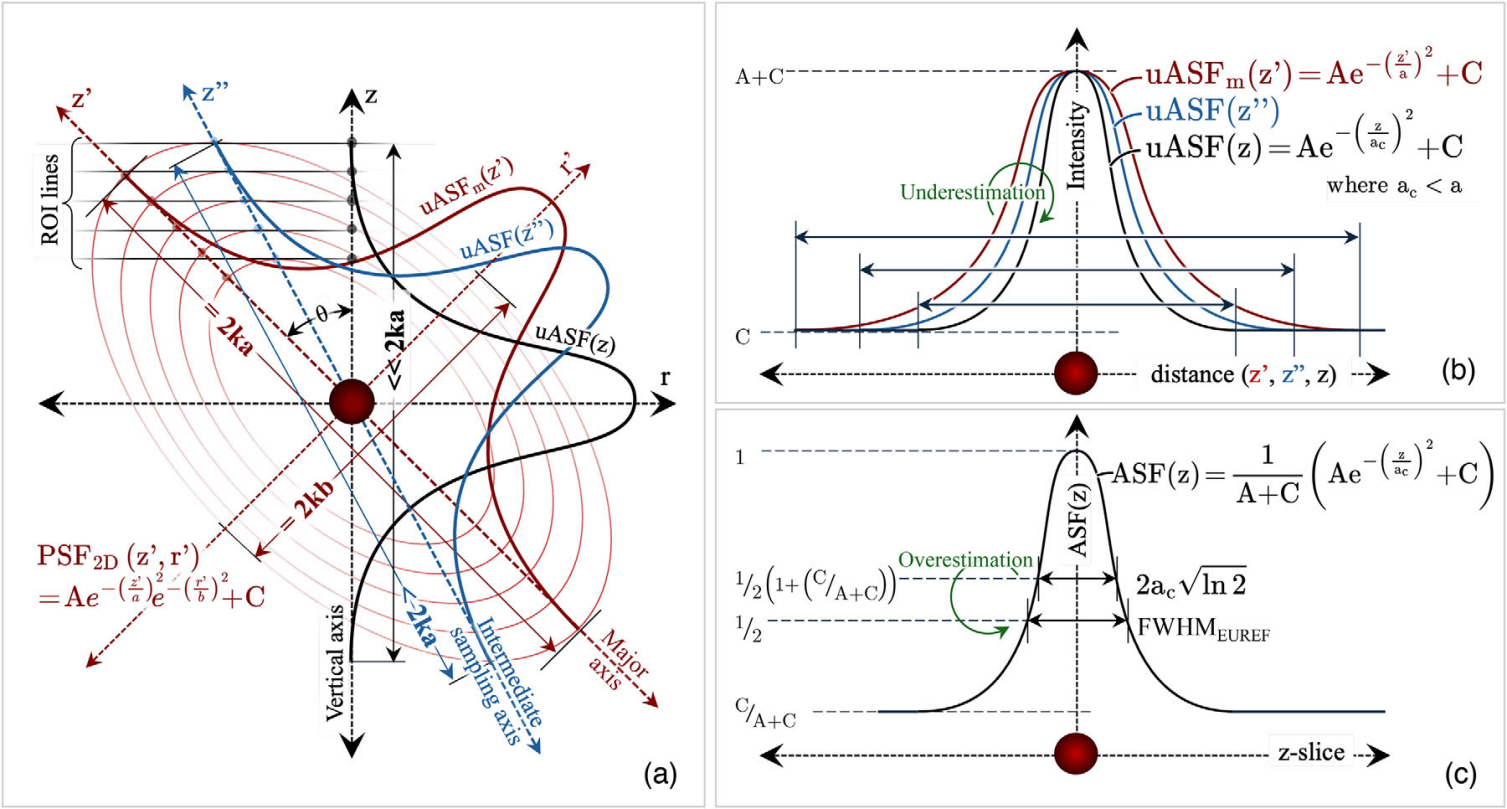
Schematic illustrating the origins of systematic errors in FWHM computed using the EUREF protocol. (a) Cross‐sectional contour view of an oblique PSF, showing the sampled uASF points lie along an intermediate sampling axis (z″) between the major (z′) and the vertical (z) axes. This is because at no slice (z) are the points along the major axis (z′) ever sampled, since they are never the points of maximum signal within the horizontal ROI lines. Note ‘k’ is simply a large enough positive scalar such that PSF_2D_(ka, kb)∼C. (b) Original uASF_m_(z′), sampled uASF(z″) and projected uASF(z) profiles overlaid on a common abscissa to illustrate progressive contraction that underestimates the FWHM. (c) Normalizing ASF(z) without correcting for its DC baseline also overestimates the FWHM eventually.

**FIGURE A1:**
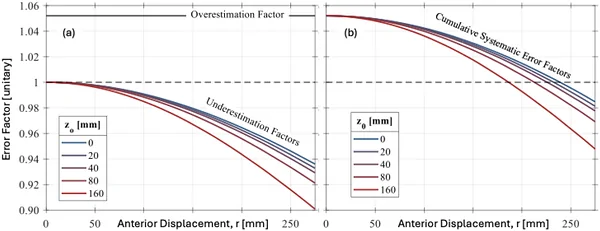
(a) Plot of over and underestimation factors versus anterior displacement for different BB elevations. (b) Cumulative systematic error factor plotted as a function of anterior displacement also for different elevations showing that even without stochastic noise, the EUREF protocol overestimates the FWHM closer to the chestwall (CW) but underestimates it away from the CW. It is this error which counter acts the expected FWHM trend against anterior displacement expected from the logic of the changing apparent angle.

Figure 2a,b demonstrates this systematic effect by examining the sagittal cross‐section of the oblique 3D PSF for an anteriorly displaced BB. In the absence of any noise, the PSF can be coarsely approximated as an ellipsoidal Gaussian with its major axis (z′) aligned toward the net direction of the X‐ray sources. In this 2D cross‐section, the planar BB‐ROIs become horizontal lines over which the maximum signal is recorded at different (z) slices per the protocol. At each maximum level, ellipsoidal contours tangent to the ROI lines are also depicted. The positions of these tangency points reveal that the uASF(z″) profile sampled lies along an intermediate axis (z″) instead of the intended major axis (z′), where it would have been uASFm(z′). This sampled uASF(z″) is then projected onto the vertical axis to express it against the abscissa of slice location (z), resulting in the final uASF(z) from which the FWHM is eventually computed. From the intended uASFm(z′) to the sampled uASF(z″) and, finally, to the projected uASF(z), there occurs a contraction at each step which makes the resulting FWHM narrower than before (Figure 2b). This effect can be quantified as a FWHM underestimation factor that can reach up to 8% for a typical DBT scan, being more severe for BBs positioned anteriorly from the CW (Appendix Figure A1).

### 2.2 Systematic overestimation of FWHM

The EUREF protocol then proceeds to compute the FWHM by evaluating the width at the intensity value midway between the peak BB signal $I_{\mathrm{BB}}^{\mathrm{RO}I_{\max}}\left(z_{0}\right)$ and the mean Bgd signal $I_{\mathrm{Bgd}}^{\mathrm{RO}I_{\mathrm{avg}}}\left(z_{0}\right)$. Equivalently, this intensity corresponds to the value of 0.5 in the normalized ASF(z). This approach however is valid only when uASF(z), and therefore ASF(z) as well, is devoid of any baseline component. When a baseline is present, however, the true half‐maximum intensity threshold lies above the absolute level of 0.5, ultimately resulting in a systematically overestimated FWHM measurement (Figure 2c).

This baseline in the sampled uASF(z) may arise from two sources. First, in simple back‐projection (SBP) reconstructions, the baseline is the contribution from a single projection, since the PSF's major axis nearly always passes through one of the 15 X‐ray sources. This implies a persistent baseline of at least 1/15th the peak BB signal in uASF(z), which, if ignored, can lead to an overestimation of FWHM by approximately 5% (Appendix Figure A1). The second contributor is stochastic noise within the reconstruction volume, which elevates the uASF(z) floor through variance‐driven effects, further distorting the FWHM estimate.

**FIGURE 3:**
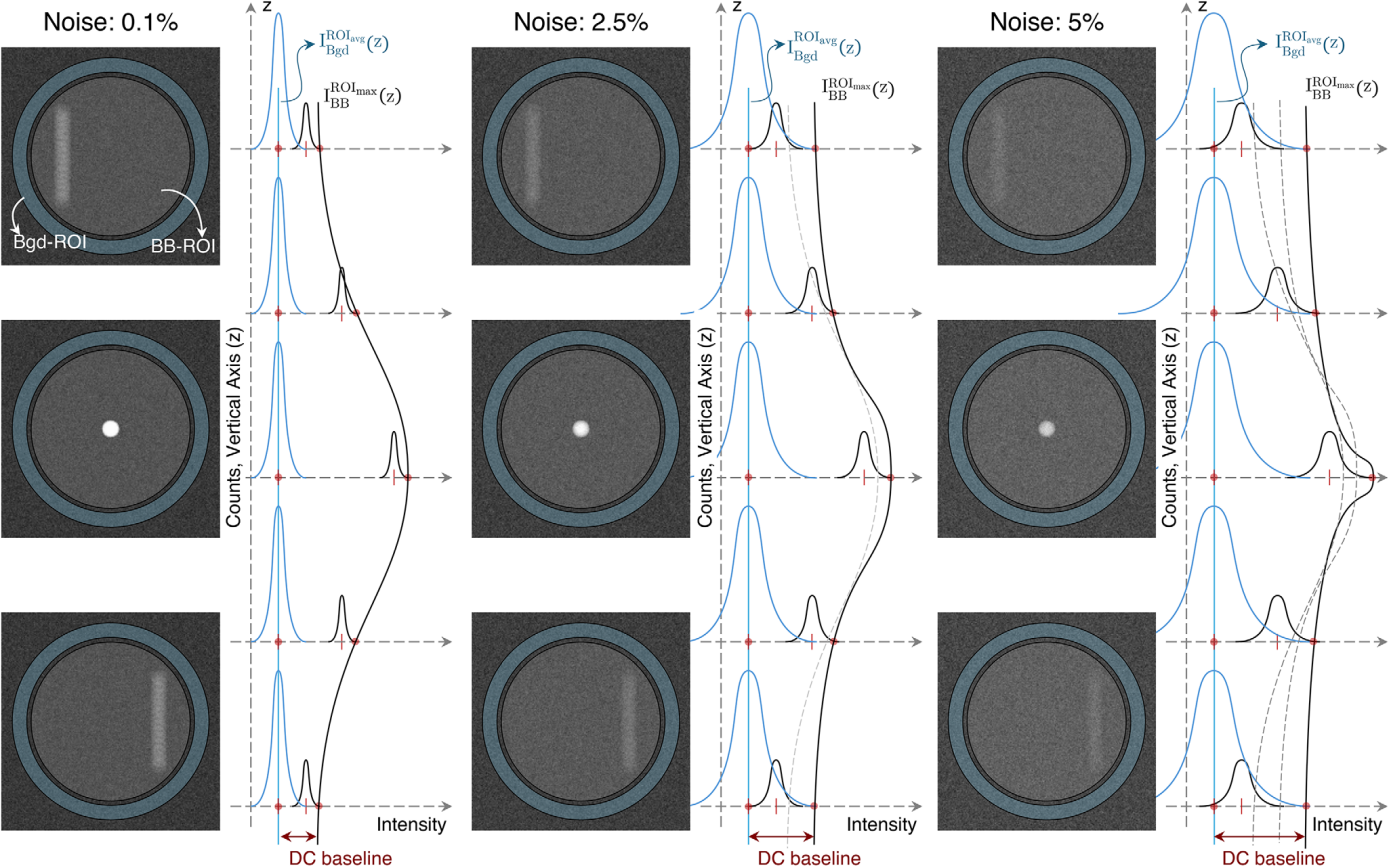
Schematic illustrating how ASF(z) systematically acquires a baseline due to stochastic noise. Reconstructions of a BB at three increasing noise levels demonstrate how noise affects the histograms of BB‐ and background‐ (Bgd) ROIs. As more purely stochastic noise in introduced, the tails of the distributions extend outward while their centers remain unchanged, introducing a baseline in and altering its overall shape in a noise‐dependent manner that ultimately affects the FWHM.

Figure 3 illustrates how introducing noise in the reconstruction volume causes the uASF(z) to progressively acquire a baseline in more detail. It considers three reconstructions of the same BB at increasing levels of zero‐mean stochastic noise. In the least noisy case on the left, histograms of the bright BB pixels and dark Bgd pixels form distinct sharp peaks. As the z‐slices vary, the separation between the peaks’ centers changes. The centers of the Bgd distribution and the right‐tail ends of the BB distribution give the $I_{\mathrm{Bgd}}^{\mathrm{RO}I_{\mathrm{avg}}}\left(z\right)$ and $I_{\mathrm{BB}}^{\mathrm{RO}I_{\max}}\left(z\right)$ profiles respectively, the difference of which is the uASF(z). When more zero‐mean noise is injected, the distributions’ tails extend outward, while their centers remain unaffected. This causes $I_{\mathrm{BB}}^{\mathrm{RO}I_{\max}}\left(z\right)$ to inevitably adopt a systematic baseline, approximately proportional to the noise's standard deviation, while also altering its shape. These changes propagate into the ASF(z) from which the FWHM is ultimately computed. Consequently, the protocol ends up reporting an artificially inflated value of FWHM under conditions of higher stochastic noise, such as from lower exposure (mAs), even between identical DBT acquisition geometries.

## 3 METHOD

### 3.1 Modified protocol

**FIGURE 4:**
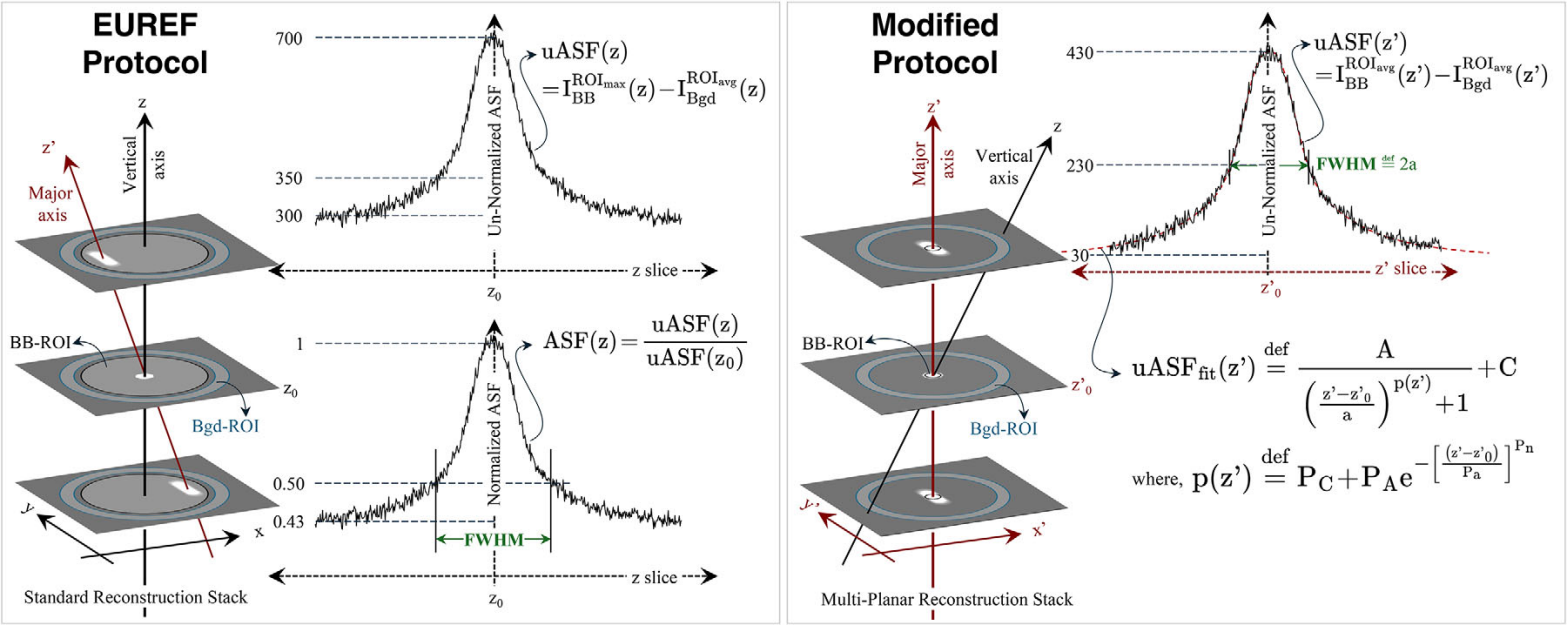
Schematic of the (a) EUREF and the proposed (b) Modified protocols. Numerical values are only for representative purpose.

The systematic dependencies inherent in the EUREF formulation motivate the development of an alternative approach for estimating z‐resolution that more faithfully reflects the local PSF structure. The proposed Modified protocol samples the tilted 3D PSF along its major axis, while remaining robust to stochastic noise in the reconstruction. It employs a multi‐planar reconstruction (MPR) view to orient the slices along the major axis of the 3D PSF such that BB signal does not blur laterally between adjacent MPR slices. It computes the uASF as the difference between the means over a small BB‐ROI and a corresponding Bgd‐ROI, within each MPR slice. This uASF, plotted against its natural abscissa of the MPR slice (z′), is then used to compute the FWHM by fitting a parameterized function uASFfit(z′) (Figure 4b) to account for any baseline in it.

The Modified protocol requires a priori determination of the MPR orientation and the functional form to fit the uASF(z′) profile with. For the former, we estimate the direction of the 3D PSF's major axis (z′) by averaging the direction vectors of all X‐ray sources relative to the BB's spatial coordinate (Figure 1b). This direction corresponds to the axis along which out‐of‐plane artifacts are most extended, reflecting the dominant direction of PSF delocalization imposed by the limited‐angle acquisition in DBT. For the fit for uASF(z′) profile, we adopt a modified Lorentzian form, uASFfit(z′) (Figure 4b), which was found to be suitable for SBP reconstructions in this study. Standard functional forms such as Gaussian and Lorentzian profiles were also evaluated but were found to either decay too rapidly or too slowly, respectively, to adequately capture the uASF(z′) falloff effectively.

We hypothesize that sampling the 3D PSF along its major axis using a PSF‐aligned MPR view per the Modified protocol will preserve the natural spatial scaling of the uASF(z′) profile, preventing it from introducing systematic underestimation of its FWHM and restore its correlation with the apparent angle. Additionally, we hypothesize that computing the uASF(z′) as the difference between the means of the BB and Bgd ROIs will render the FWHM measurement more robust against bias from stochastic noise.

**Table:** 

Pseudocode for Modified Protocol	
**Input**: Reconstruction volume $V(\vec{r})$ defined on $\vec{r}=\left(\hat{x},\hat{y},\hat{z}\right)$ BB location $\vec{B}$; BB's radius $r_{\mathrm{BB}}$; Source positions ${\left\{{\vec{S}}_{i}\right\}}_{i=1}^{15}$	
**Output**: FWHM at $\vec{B}$	
1.	**Crop BB‐centered VOI**: $V\left(\vec{r}\right)\overset{\mathrm{crop}\;\mathrm{around}\;\vec{B}}{\to }V_{\mathrm{VOI}}\left(\vec{r};\vec{B}\right)$
2.	**Compute PSF's major axis** $\left(\hat{z^{\prime }}\right)$ **for MPR view**: $${\vec{z}}^{\prime }\overset{\text{def}}{=}\frac{1}{15}\sum_{i=1}^{15}\frac{{\vec{\mathrm{BS}}}_{i}}{{∥\vec{\mathrm{BS}}∥}_{i}},\;\mathrm{where}\;{\vec{\mathrm{BS}}}_{i}\overset{\text{def}}{=}{\vec{S}}_{i}-\vec{B},\;\hat{z^{\prime }}=\frac{{\vec{z}}^{\prime }}{∥{\vec{z}}^{\prime }∥}$$
3.	**Resample & reorient VOI into MPR coordinates**: $\vec{r^{\prime }}=\left(\hat{x^{\prime }},\hat{y^{\prime }},\hat{z^{\prime }}\right)$: $V_{\mathrm{VOI}}\left(\vec{r};\vec{B}\right)\to V_{\mathrm{VOI}}^{MPR}\left(\vec{r^{\prime }};\vec{B}\right)$
4.	**Draw 2D ROIs** $\Omega \subset R^{2}$ **in the** $\hat{x^{\prime }},\hat{y^{\prime }}$ **‐plane on each** $z^{\prime }$ **slice**:
	BB‐ROI $\left(\Omega_{\mathrm{BB}}\right)$ = circle of small radius $r\in \left(0,\frac{r_{\mathrm{BB}}}{2}\right)$ centered about BB $\left(\vec{r^{\prime }}=0\right)$ Background‐ROI $\left(\Omega_{\mathrm{Bgd}}\right)$ = ROI nearby excluding the BB signal
5.	**Compute the un‐normalized ASF(z′)**: $\mathrm{uASF}\left(z^{\prime }\right)\overset{\text{def}}{=}I_{\mathrm{BB}}^{\mathrm{RO}I_{\mathrm{avg}}}\left(z^{\prime }\right)-I_{\mathrm{Bgd}}^{\mathrm{RO}I_{\mathrm{avg}}}\left(z^{\prime }\right)$ $$\begin{array}{c} I_{\mathrm{BB}}^{\mathrm{RO}I_{\mathrm{avg}}}\left(z^{\prime }\right)\overset{\text{def}}{=}\frac{1}{|\Omega_{\mathrm{BB}}|}\sum_{\left({\vec{x}}^{\prime },{\vec{y}}^{\prime }\right)\in \Omega_{\mathrm{BB}}}V_{\mathrm{VOI}}^{MPR}\left(\vec{r^{\prime }};\vec{B}\right),\\ I_{\mathrm{Bgd}}^{\mathrm{RO}I_{\mathrm{avg}}}\left(z^{\prime }\right)\overset{\text{def}}{=}\frac{1}{|\Omega_{\mathrm{Bgd}}|}\sum_{\left({\vec{x}}^{\prime },{\vec{y}}^{\prime }\right)\in \Omega_{\mathrm{Bgd}}}V_{\mathrm{VOI}}^{MPR}\left(\vec{r^{\prime }};\vec{B}\right) \end{array}$$
6.	**Fit** $\mathrm{uASF}(z^{\prime })$ **to** $\mathrm{uAS}F_{\mathrm{fit}}\left(z^{\prime }\right)$ **and obtain the FWHM**: 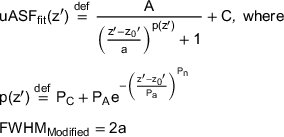

### 3.2 Analytical simulation

We simulate the SBP reconstruction of a spherical BB imaged by a DBT acquisition using an analytical formulation. The method backprojects the Radon transform of an ideal sphere in free space yielding a closed‐form expression which is evaluated over all voxels to obtain the reconstruction [Appendix §D]. This simulation produces a noiseless 3D tomographic SBP reconstruction. We progressively corrupt this reconstruction by adding a zero‐mean Gaussian noise of known variance *post hoc* to introduce controlled stochastic variability. BBs are modeled as solid spheres distributed at various locations along the midplane, extending anteriorly from the CW and superiorly from the breast support. The DBT geometry of our in‐house Next‐Generation Tomosynthesis (NGT) prototype is simulated [Table 1].^11^

**TABLE 1:** Methods summary.

Attribute	Analytical Simulation	Physical Imaging
**BB Phantom**	1 mm solid spheres in free space	1 mm steel spheres between 2.5 cm thick PMMA slabs
**BB Displacement**	Anteriorly: 0–250 mm Superiorly: 0–100 mm	Anteriorly: 0–225 mm
**Stochastic Noise Source**	Added post‐hoc (zero‐mean Gaussian)	Inherent from exposure
**Noise Range** ^a^ **/ Technique**	0%–7.5%	1.8%–6% 30 kV | 100–10mAs
**Imaging Geometry**	NGT prototype, SID = 762.5 mm, Angular span = 15°	
**Reconstruction Method**	Simple Back‐Projection (SBP)	
**Reconstruction Resolution**	0.08 × 0.08 × 0.08 mm	0.08 × 0.08 × 0.20 mm

^a^ Noise level [%] expressed as standard deviation relative to peak BB contrast. Higher noise levels were achieved by reducing the mAs for physical imaging.

A simple, spatially invariant, zero‐mean Gaussian noise model was intentionally employed to isolate the influence of purely stochastic noise on z‐resolution trends. Real‐world reconstruction algorithms may exhibit spatially varying noise patterns, for example with distance from the chest wall, which can introduce position‐dependent confounds and were therefore excluded from this controlled analysis examining the sole effect of adding noise on the spatial trends of FWHM.

The simulation framework enabled quantification of both the systematic and random dependencies present in the protocols. Specifically, we compared their ability to capture trends in z‐resolution arising from the narrowing and widening of apparent angle expected with anterior and superior displacements, respectively. Noise‐free reconstructions were used to isolate intrinsic trends, and the noise‐laden reconstructions to assess robustness to stochastic variability. To quantify systematic noise dependence, FWHM‐versus‐displacement trends for each protocol were fit to polynomials. The resulting regression parameters (intercept, goodness‐of‐fit R^2^) were analyzed as functions of noise level to assess bias and robustness. To evaluate conformity with the geometry of the local PSF, we also examined the FWHM‐versus‐apparent angle trends and performed linear regressions. The resulting parameters (slopes, R^2^) were analyzed across noise levels to assess preservation of the expected inverse relationship. Significance of regression coefficients was assessed using t‐tests. For R^2^ values between‐protocol differences were assessed using the Wilcoxon rank‐sum test. Where multiple comparisons were made across noise levels, p‐values were corrected using the Holm method to control the family‐wise error rate. Statistical significance was assessed at the 0.05 level.

Additionally, since the Modified protocol samples the BB signal using the mean of a small ROI, the choice of ROI size may inadvertently introduce secondary systematic effects. To evaluate this potential sensitivity, we varied the BB‐ROI dimensions, ceteris paribus, and examined the stability of the resulting FWHM estimates. This analysis allowed identification of a suitable range of BB‐ROI sizes within which the Modified protocol remains sufficiently robust.

### 3.3 Physical imaging

To physically validate the Modified protocol's ability to capture the narrowing of apparent angle with anterior displacement, we applied it to reconstructions of a custom‐built phantom imaged on our NGT DBT system.^11^ The phantom contains steel BBs spaced ∼15 mm apart, spanning an anterior range of 225 mm from the CW. We imaged the phantom at multiple mAs settings to introduce varying levels of stochastic noise in the reconstruction (Table 1). The reconstructions were generated at a slice spacing of 0.2 mm using Piccolo™ (Real Time Tomography LLC, Villanova, PA). We applied both protocols to examine their ability to preserve expected trends and reduce noise‐induced bias. A custom MATLAB script was used to manually locate the BBs and extract volumes centered on each. For the Modified protocol, these volumes were rotated using known X‐ray source coordinates and BB positions to generate MPR views aligned with the expected PSF's major axis (z′). The EUREF protocol employed the unrotated (non‐MPR) volumes. Similar regression analyses were performed on the experimental data to evaluate (i) systematic dependence on noise level and (ii) preservation of the expected anti‐linearity between FWHM and apparent angle under physical imaging conditions.

## 4 RESULTS

### 4.1 Simulations: FWHM versus anterior displacement

**FIGURE 5:**
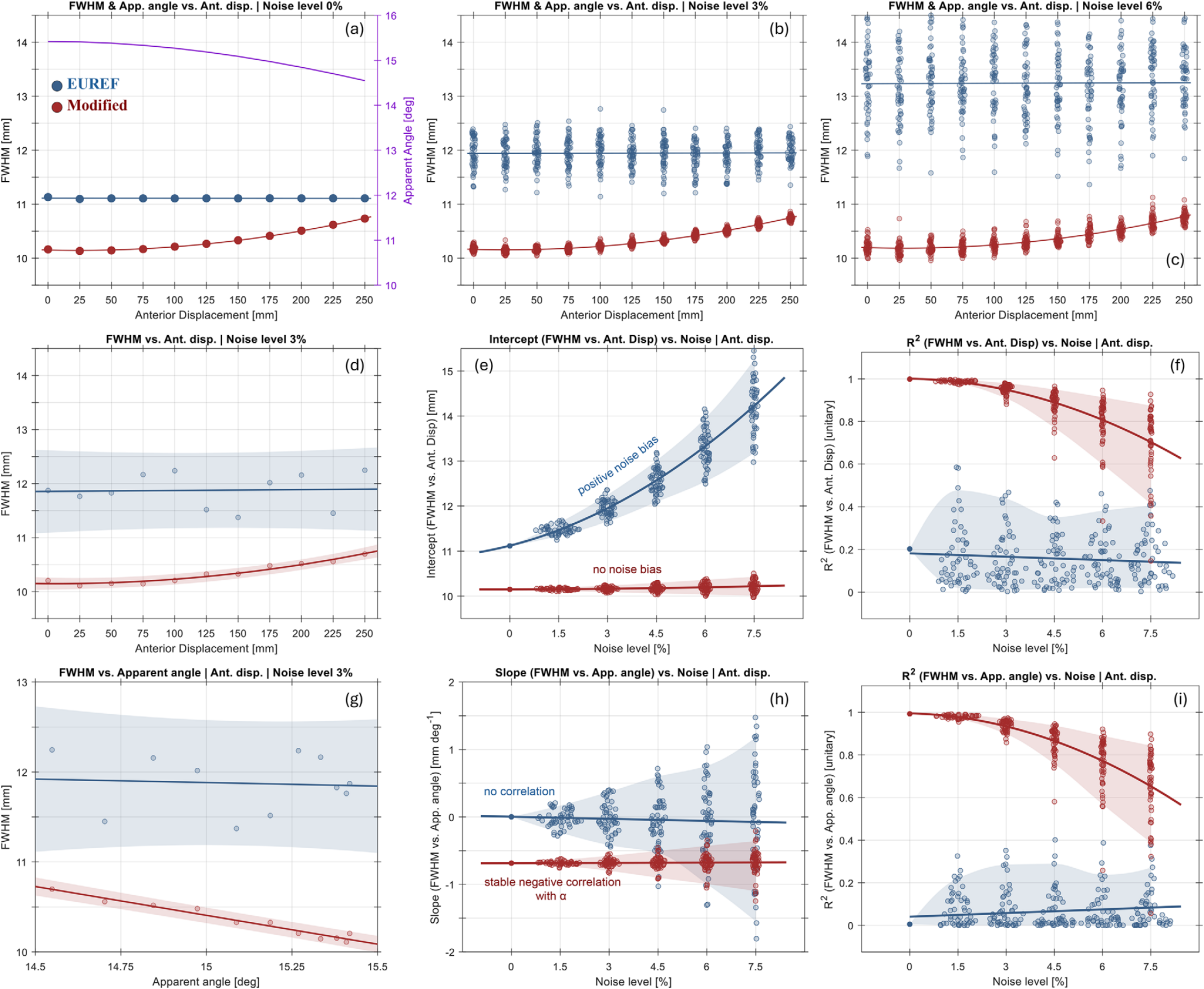
Simulation: Anterior Displacement. FWHM behavior under anterior BB displacement for the EUREF (blue) and Modified (red) protocols across noise levels. Top Row (a–c): FWHM versus anterior displacement at 0%, 3%, and 6% noise (50 realizations per position). (a) also shows the corresponding apparent angle (purple, right axis). At 0% noise, the Modified protocol increases with displacement, consistent with narrowing of the apparent angle, whereas EUREF remains approximately flat. With increasing noise (b–c), EUREF shifts upward, indicating systematic bias, while the Modified protocol preserves the expected spatial trend with substantially reduced drift. Solid curves denote the regression polynomial fits (quadratic for Modified; linear for EUREF). Middle Row (d–f): Quantification of noise bias. (d) Representative realization at 3% noise illustrating the fitting procedure used to extract intercept and R^2^ for each protocol. (e) Intercepts versus noise (50 realizations per level) show a positive bias for EUREF and near‐constant behavior for Modified, indicating reduced noise sensitivity. (f) Corresponding R^2^ values begin near unity for Modified and decline with increasing variance, whereas EUREF R^2^ values remain low across all noise levels, reflecting poor displacement‐trend capture. Bottom Row (g–i): FWHM versus apparent angle and linear‐correlation analysis. (g) Representative realization at 3% noise demonstrating a clear negative linear trend for Modified and weak correlation for EUREF. (h) Slopes of FWHM versus apparent angle (50 realizations per level) remain consistently negative for Modified but cluster near zero for EUREF. (i) R^2^ values of the linear regressions begin near unity for Modified and degrade with noise, remaining substantially higher than EUREF, whose R^2^ remains low, indicating weak geometric coupling between its FWHM and apparent angle. Note (b,c,e,f,h,i) are swarmplots.

Figure 5 presents the simulated FWHM trends as a function of anterior BB displacement. In the noise‐free case (Figure 5a), the EUREF protocol depicts a near constant FWHM with anterior displacement, failing to capture the sworsening of z‐resolution associated with the progressive narrowing of the apparent angle. In contrast, the Modified Protocol demonstrates a clear monotonic increase in FWHM with anterior displacement, consistent with the variation in the apparent angle. When stochastic noise is introduced (Figure 5b,c), the two protocols exhibit divergent behavior. The EUREF protocol exhibits an upward shift in FWHM values with increasing noise level, whereas the Modified protocol maintains comparatively stable estimates.

To quantify this noise dependency, regression models were fitted to the FWHM‐displacement data. The intercepts were analyzed as a function of noise to assess bias, and the associated goodness‐of‐fit metrics (R^2^) were monitored. The EUREF protocol demonstrated a strong positive correlation between intercept and noise (bias = +0.46 mm/%; *p* < 10^−15^), whereas the Modified protocol exhibited a markedly reduced dependence (bias = +0.01 mm/%; *p* = 0.15). The Modified protocol reduced the systematic noise‐induced bias by 98% (*p* < 10^−15^) relative to the EUREF protocol (Figure 5e). Fit quality (R^2^, FWHM‐displacement) for the Modified protocol began at unity and degraded with increasing noise but remained consistently above EUREF's for the noise levels tested (Figure 5f).

To evaluate concordance with the local PSF geometry, FWHM was plotted against apparent angle and linear regressions were performed. The Modified protocol exhibited a stable negative slope across noise levels (mean slope = −0.68 mm·deg^−^^1^), consistent with an inverse linear relationship between FWHM and apparent angle. In contrast, the EUREF protocol yielded near zero slopes (mean slope = −0.002 mm·deg^−^^1^), suggesting weak or absent correlation with apparent angle (Figure 5h). Slopes differed significantly between protocols (*p* < 10^−15^) at all noise levels. Corresponding R^2^ (FWHM–angle) values were substantially higher for the Modified protocol (overall median *R^2^* = 0.885 vs. 0.034), with differences significant at all tested noise levels (Holm‐corrected Wilcoxon tests, all noise levels *p* < 10^−15^). (Figure 5i).

### 4.2 Simulations: FWHM versus superior displacement

**FIGURE 6:**
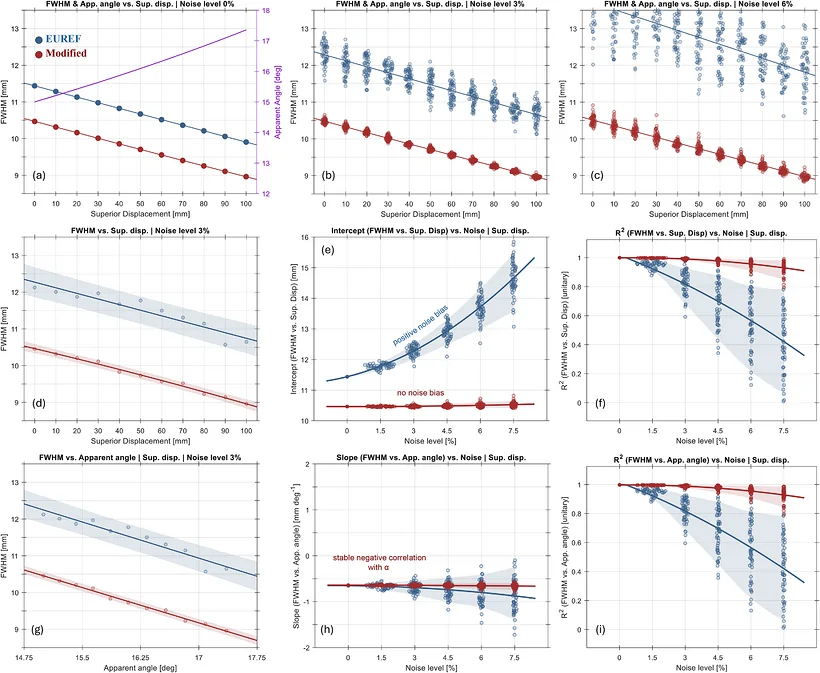
Simulation: Superior Displacement. FWHM behavior under superior BB displacement for the EUREF (blue) and Modified (red) protocols across noise levels. Top Row (a–c): FWHM versus superior displacement at 0%, 3%, and 6% noise (50 realizations per position). (a) also shows the corresponding apparent angle (purple, right axis). At 0% noise, both protocols’ FWHM's descrease with displacement, consistent with widening of apparent angle superiorly. With increasing noise (b–c), EUREF shifts upward, indicating systematic bias unlike the Modified protocol. Solid curves denote the linear polynomial fits. Middle Row (d–f): Quantification of noise bias. (d) Representative realization at 3% noise illustrating the fitting procedure used to extract intercept and R^2^ for each protocol. (e) Intercepts versus noise (50 realisations per level) show positive bias for EUREF and near‐constant behavior for Modified, indicating reduced noise sensitivity. (f) Corresponding R^2^ values start near unity for both protocols at low noise; however, R^2^ for EUREF degrades more rapidly with increasing noise. Bottom Row (g–i): FWHM versus apparent angle and linear‐correlation analysis. (g) Representative realization at 3% noise showing a negative linear trend for both protocols albeit with greater variability for the EUREF case (h) Slopes of FWHM versus apparent angle (50 realisations per level) remain negative for both protocols, with EUREF exhibiting greater variability for higher noise levels. (i) R^2^ values of the linear regressions start near unity for both but they degrade fasters with noise for the EUREF protocol, indicating weak coupling between its FWHM and apparent angle. Note (b,c,e,f,h,i) are swarmplots.

For superior displacement, both protocols exhibited decreasing FWHM with superior displacement in the noise‐free case, consistent with the widening of apparent angle (Figure 6a), indicating that when PSF obliquity is minimal, both protocols capture the variation in apparent angle. However, with increasing noise (Figure 6b,c,e), the EUREF protocol again demonstrated a positive bias in FWHM (bias = +0.48 mm/%; *p* < 10^−15^), whereas the Modified protocol remained comparatively stable (bias = +0.01 mm/%; *p* = 0.18). Once again, the Modified protocol reduced the systematic noise‐bias by 98% (*p* < 10^−15^) relative to the EUREF protocol. Here, both protocols retained a negative slope in the FWHM‐apparent angle linear regression at −0.64 mm deg^−^^1^ (Figure 6h) although the Modified protocol held this value more consistently for higher noise. Fit quality (R^2^, FWHM–angle) began at unity for both protocols and degraded with increasing noise but, remained higher for the Modified protocol (Holm‐corrected Wilcoxon tests, all noise levels *p* < 10^−15^), indicating greater preservation of the anti‐linearity with apparent angle despite stochastic degradation (Figure 6i).

### 4.3 Simulations: origin of systematic bias in EUREF

**FIGURE 7:**
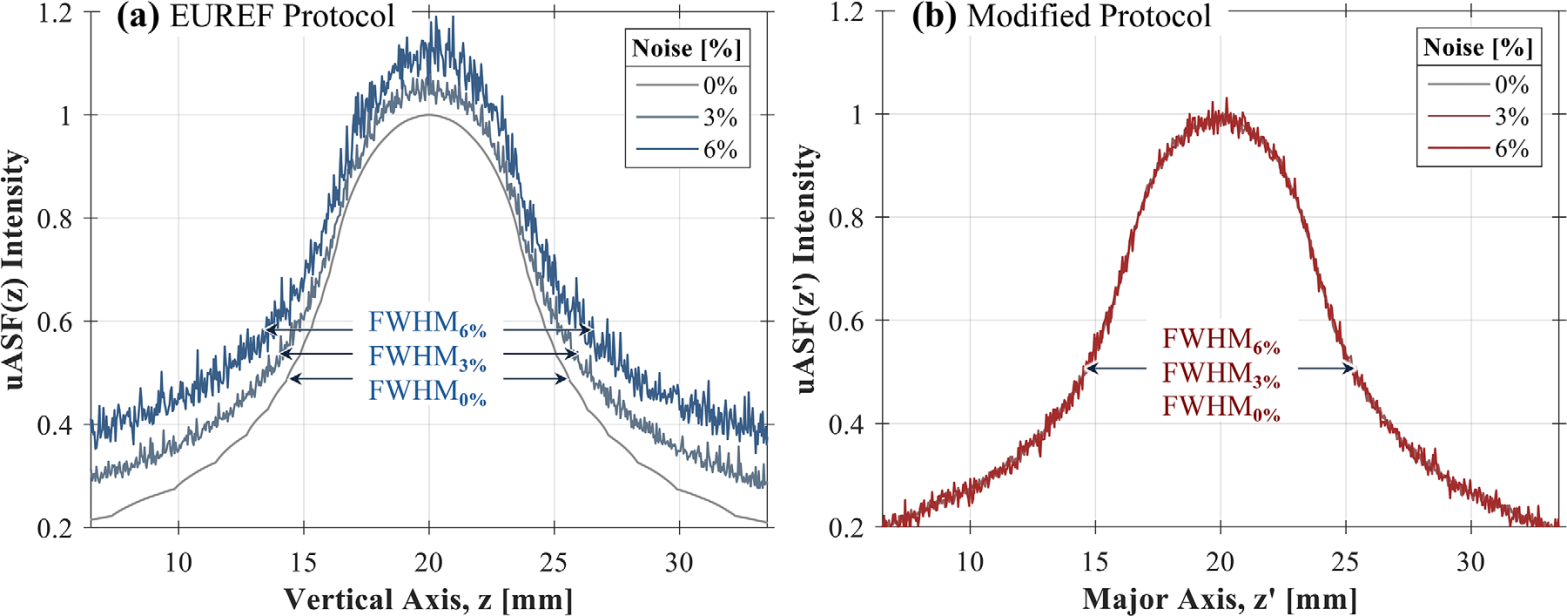
Impact of stochastic noise on uASF(z) profiles under the EUREF and Modified protocols using simulated data. (a) EUREF protocol shows a systematic noise‐dependent positive bias, which widens the uASF(z) profile and introduces a DC baseline in it, ultimately resulting in an overestimation of FWHM. (b) In contrast, the Modified protocol's uASF(z′) profiles remain superimposed on each other across all noise levels, demonstrating minimal systematic bias due to stochastic noise.

To elucidate the origin of the positive systematic bias in the EUREF protocol, uASF profiles derived from both protocols were compared *in toto* (Figure 7). The EUREF uASF(z) was found to exhibit a progressive baseline elevation with increasing noise, consistent with the histogram analysis in Figure 3. The resulting FWHM inflation arises from failure to account for this noise‐induced offset as indicated in Figure 2c. In contrast, the Modified Protocol's uASF(z′) maintains a constant baseline irrespective of noise levels, explaining its greater systematic stability (Figure 7b).

### 4.4 Simulations: FWHM versus BB‐ROI radius

**FIGURE 8:**
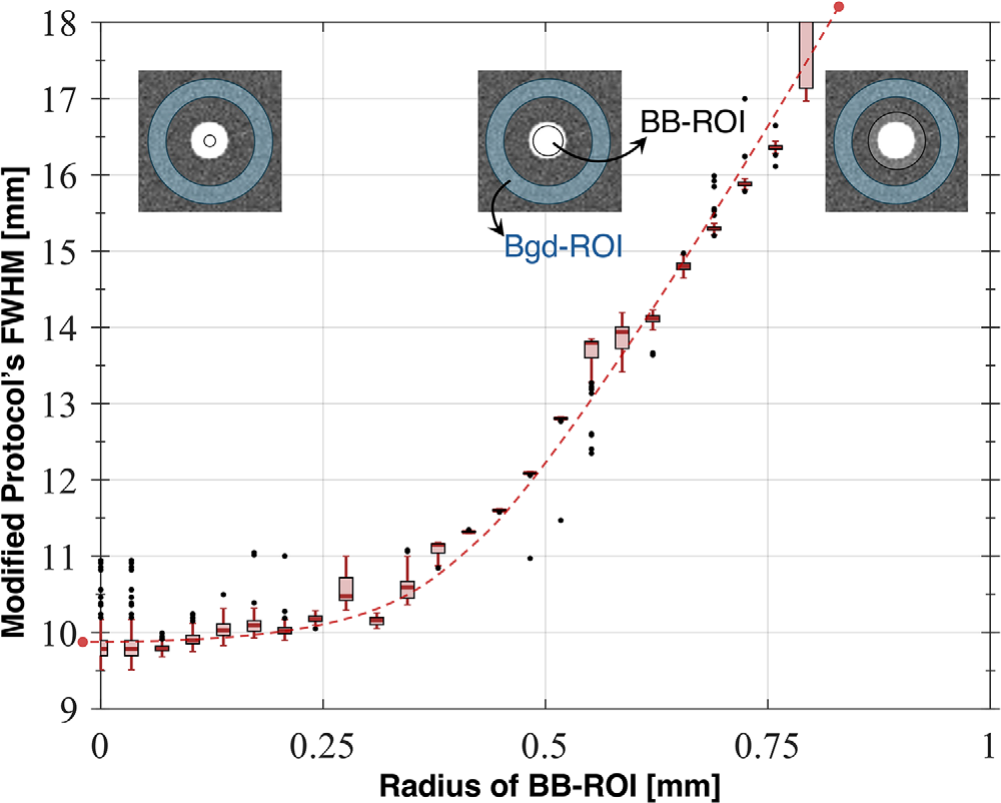
Modified protocol's FWHM versus radius of BB‐ROI. At small radii the trend plateaus while at larger radii the FWHM increases. The increase occurs because after the ROI exceeds the physical dimensions of the BB (radius 0.5 mm), profile incorporates more unwanted background signal, leading to a flattened uASF(z′). A smooth fit (dashed red line) may be used to estimates the asymptotic FWHM at infinitesimally small ROI size to provide a more stable and robust measurement. Insets illustrate ROI sizes relative to the BB.

As outlined in Methods, the Modified protocol requires the BB‐ROI to be sufficiently small such that its mean intensity reflects only the BB signal, avoiding contamination from neighboring background pixels. Here, (Figure 8) we report the corresponding sensitivity analysis evaluating how FWHM varies with increasing BB‐ROI size. FWHM increased with ROI radius, as larger ROIs incorporate more background signal, flattening the uASF(z′) profile and broadening the measured FWHM. When the ROI radius was maintained below half the physical BB radius (< 0.25 mm), FWHM remained stable. Thus, we recommend a radius of ∼1/4 the BB radius as a stable operating point for practical implementation, balancing stochastic averaging (larger BB‐ROI) against exclusion of background signal (smaller BB‐ROI).

### 4.5 Physical reconstruction: Anterior displacement

**FIGURE 9:**
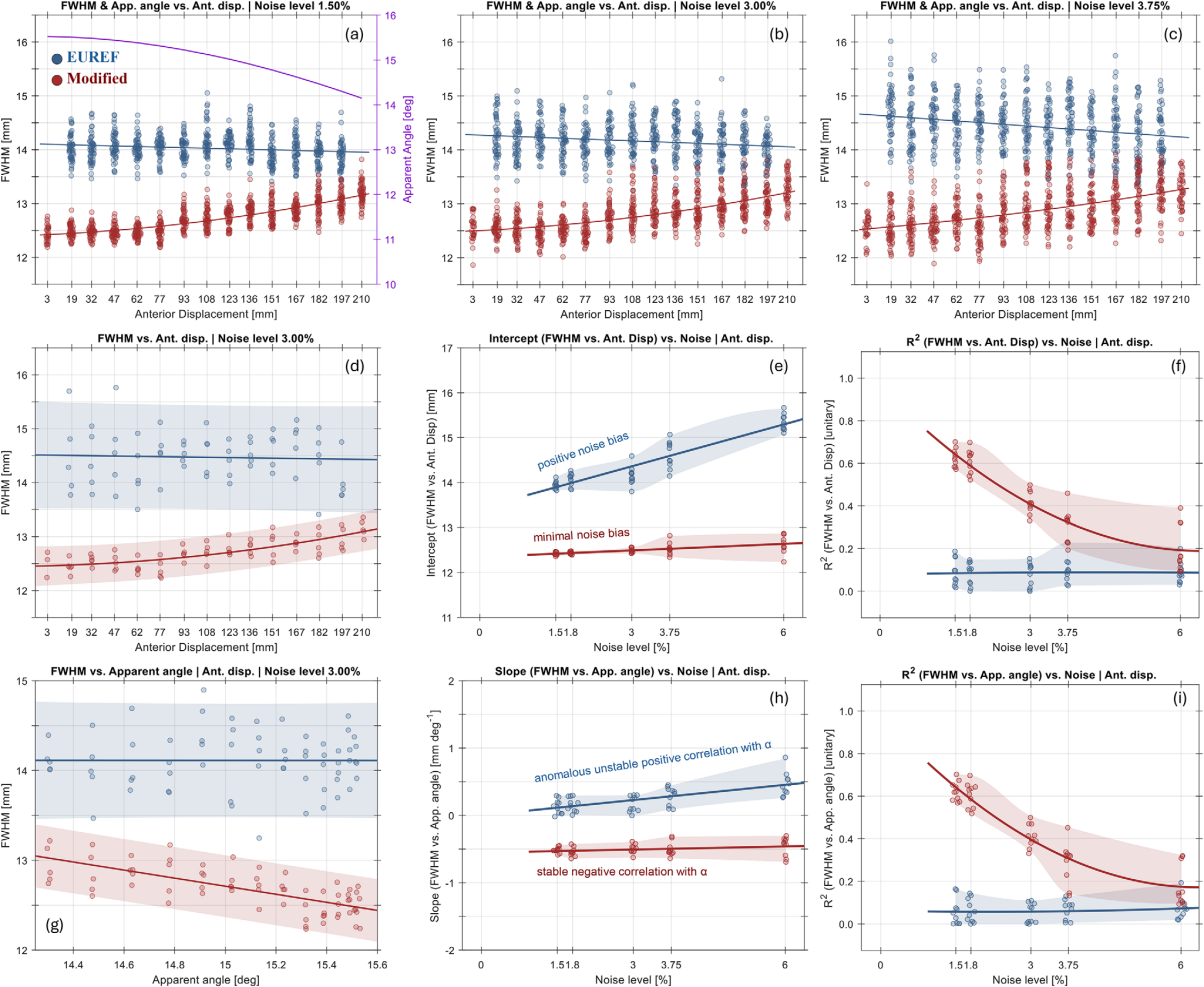
Physical Imaging: Anterior Displacement. Experimental FWHM behavior under anterior BB displacement for the EUREF (blue) and Modified (red) protocols across noise levels. Top Row (a–c): FWHM versus anterior displacement at 1.5%, 3%, and 3.75% noise (50 realizations per position). (a) also shows the corresponding apparent angle (purple, right axis). The Modified protocol exhibits a gradual increase in FWHM with displacement, consistent with narrowing of the apparent angle, whereas EUREF remains comparatively flat and progressively shifts upward with increasing noise, indicating systematic bias. Solid curves denote the regression polynomial fits (quadratic for Modified; linear for EUREF). Middle Row (d–f): Quantification of noise bias. (d) Representative realization at 3% noise illustrating the fitting procedure used to extract intercept and R^2^ for each protocol. (e) Intercepts versus noise (10 realizations per level) show a modest positive drift for the Modified protocol but a substantially steeper positive bias for EUREF, indicating reduced yet non‐zero noise sensitivity for Modified relative to EUREF. (f) Corresponding R^2^ values do not originate at zero‐noise conditions but are highest at the lowest measured noise (1.5%); R^2^ for Modified remains consistently higher and declines gradually with noise, whereas EUREF R^2^ values remain comparatively low across all noise levels, reflecting poor displacement‐trend capture. Bottom Row (g–i): FWHM versus apparent angle and linear‐correlation analysis. (g) Representative realization at 3% noise demonstrating a clear negative linear trend for Modified and weaker, more dispersed behavior for EUREF. (h) Slopes of FWHM versus apparent angle (10 realizations per level) remain stably negative for Modified across noise levels, while EUREF slopes exhibit greater variability and are near zero or paradoxically positive at higher noise levels. (i) R^2^ values of the linear regressions are consistently higher for Modified and decline gradually with noise, whereas EUREF R^2^ values remain low throughout, indicating weak geometric coupling between its FWHM and apparent angle in the physical imaging case. Note (b,c,e,f,h,i) are swarmplots.

Physical reconstructions of the custom‐built phantom containing anteriorly displaced BBs yielded trends consistent with simulation findings (Figure 9). In the lowest‐noise condition, Modified protocol demonstrated an increasing FWHM with anterior displacement, consistent with apparent angle narrowing, whereas the EUREF protocol remained comparatively flat (Figure 9a). With increasing noise (via reduced mAs), the EUREF protocol once again exhibited a positive bias in FWHM, while the Modified Protocol remained more stable (Figure 9b,c). Regression‐based intercept analysis (FWHM‐displacement) confirmed that noise‐induced bias was markedly smaller with the Modified protocol (bias = +0.15 mm/%; *p* = 0.002) than with EUREF (bias = +0.93 mm/%; *p* < 10^−15^), representing a ∼84% (*p* < 10^−20^) reduction (Figure 9e). Linear regression of FWHM‐apparent angle further demonstrated preservation of the expected inverse relationship under the Modified protocol (mean slope = −0.50 mm deg^−^^1^; *p* < 10^−15^), whereas the EUREF protocol showed a positive mean slope (+0.24 mm deg^−^^1^), suggesting a paradoxical reversal of the relationship with the apparent angle (Figure 9h). Slopes differed significantly between protocols (*p* < 10^−4^) across all noise levels. Corresponding R^2^ values (FWHM‐angle) were significantly higher for the Modified protocol at all noise levels (Holm‐corrected Wilcoxon rank‐sum tests, all noise levels *p* < 0.002) even as they, expectedly, declined with noise (Figure 9i). These findings support that the Modified protocol substantially mitigates noise‐induced bias and preserves anti‐linearity with the apparent angle.

## 5 DISCUSSION

Z‐resolution in DBT is inherently spatially variant and the Modified protocol introduced here is uniquely positioned to reveal this. While the EUREF protocol remains the prevailing standard for estimating z‐resolution, our findings demonstrate that it is prone to systematic biases that limit its ability to reflect spatial variations in the local PSF across the reconstruction volume. It exhibits two competing biases: a geometric underestimation of FWHM arising from PSF obliquity and the reliance on max‐based ASF sampling, and a noise‐dependent overestimation driven by a FWHM computation method which ignores the baseline elevation in the uASF profile.

The geometric underestimation (Figure 2c) stems from the fact that the uASF(z) signal, extracted as the slice‐wise maximum within the BB‐ROI, does not sample the true major axis of the tilted 3D PSF. Instead, it samples a profile oriented in between the true major axis and the vertical axis. Expressing this sampled profile against the vertical (z) slice coordinate further compresses the profile along its abscissa, compounding the eventual underestimation. This effect is most pronounced for anteriorly displaced BBs, where PSF obliquity is greatest. Consequently, the EUREF protocol fails to reproduce the expected increase in FWHM associated with narrowing apparent angle anteriorly—a discrepancy that has also been reported in literature ^3^, ^4^, ^10^.

In addition, neglecting the non‐zero baseline in the EUREF‐derived uASF introduces another systematic bias. Because the uASF is constructed as a discordant subtraction between the maximum and mean, the baseline scales with noise variance. Even in the complete absence of stochastic noise, a residual baseline proportional to 1/15 of the peak signal persists in SBP reconstruction (15 projections per acquisition). This baseline contribution inflates the measured FWHM, introducing a positive bias that scales with noise in the reconstructed image.

The Modified protocol addresses both limitations. By reorienting the reconstruction volume in MPR view, it samples the PSF along its true major axis, preserving its natural spatial scaling. By constructing the uASF from mean‐based BB and background ROIs and incorporating an explicit baseline parameter in the functional fit, it substantially mitigates noise‐induced bias and reduces stochastic variability. In simulations and physical experiments, the Modified protocol restored the expected anterior trend of increasing FWHM with decreasing apparent angle and reduced systematic noise‐bias on FWHM by 98% and 84%, respectively. Collectively, these findings demonstrate it to be a more reliable metric for assessing z‐resolution in DBT systems.

A central finding of this work is the recovery of a robust linear anti‐correlation between FWHM and apparent angle when using the Modified protocol. This relationship is physically intuitive: in DBT, the effective depth‐sampling of a point object is governed by the angle subtended across the source trajectory, i.e. the apparent angle. As this angle narrows, depth localization degrades and FWHM increases. This relationship should hold not just globally—between narrow (15°) and wide (50°) DBT geometries—but also locally within a DBT geometry itself, where the PSF varies spatially. Over typical distances within the reconstruction volume, the apparent angle varies by ∼6% anteriorly and ∼13% superiorly (Figure 1c). Expanding FWHM as a function of apparent angle about its mean value, higher‐order terms scale as the square of this fractional variation contributing at most (6%^2^ =) 0.4% and (13%^2^ =) 1.7% respectively and thus rendering the first‐order linear dependence dominant over the range tested. Therefore, a linear anti‐correlation between FWHM and apparent angle is expected to prevail. The strong negative slopes (Figures 5h, 6h, 9h) and high R^2^ values (Figures 5i, 6i, 9i) observed with the Modified protocol support this first‐order behavior, whereas the near‐zero slopes (Figures 5h, 9h) and low R^2^ values (Figures 5i, 9i) obtained with the EUREF protocol in the anterior direction indicate a breakdown in this relationship. Note, although apparent angle serves as a first‐order descriptor in conventional DBT source trajectories, more complex geometries, such as next‐generation tomosynthesis (NGT) systems employing two‐dimensional source motion,^12^, ^13^, ^14^, ^15^ may naturally require the consideration of apparent solid angle. Nevertheless, nothing in the Modified protocol is inherently restricted to linear source trajectories; it can be applied to more complex two‐dimensional acquisition geometries as well.

At a more fundamental level, the distinction between the EUREF and Modified protocol is also conceptual. The EUREF protocol operationally defines z‐resolution in a manner that implicitly incorporates both deterministic PSF geometry and stochastic noise, albeit with systematic errors. As a result, the EUREF derived FWHM behaves as a composite metric influenced both by geometric structure and noise fluctuations. In contrast, the Modified protocol is designed to isolate the underlying geometry of the local PSF. The resulting FWHM is more tightly coupled to apparent angle—and therefore to the PSF structure—while substantially reducing stochastic influence. If a z‐resolution metric is intended to characterize the behavior of out‐of‐plane artifacts—that is, how rapidly a signal defocuses between adjacent slices—it is desirable to have it predominantly reflect intrinsic characteristics of the PSF, rather than noise. Although noise remains an integral component of overall image quality that naturally affects detectability, it can be quantified independently through variance‐based measures (e.g., NPS, SNR) without conflating it with the PSF structure. Just as MTF does not change with increased quantum noise, a geometry‐driven z‐resolution measure is more meaningful when it is not systematically biased by noise, as it may confound stochastic fluctuations with geometric variations affecting the PSF and thereby limiting its ability to discern genuine differences in depth resolution.

From a clinical perspective, the spatial dependence of z‐resolution described here is consistent with observed variations in image interpretability across the breast volume. Cancer detection in the periareolar and subareolar regions anteriorly is well documented to be more challenging,^16^ and simulation studies have further shown that lesion detectability is lowest in the most anterior portions of the reconstructed volume.^17^ The findings in this work provide a physical basis for these observations, demonstrating that anterior displacement, through narrowing of the apparent angle, degrades the depth resolution. A z‐resolution metric that fails to capture this spatial variation will therefore obscure clinically meaningful aspects of DBT performance. The Modified protocol, by contrast, faithfully reflects these spatial variations.

This work, however, is not without its limitations. First, the Modified protocol relies on MPR visualization, which may not be natively supported on commercial DBT systems—in which case additional processing is required to rotate the standard reconstruction stack to the desired orientation. Second, obtaining the correct MPR orientation requires a priori knowledge of the X‐ray source positions in the DBT geometry. While this information was accessible in our study through a calibration routine, it may not be readily obtainable in all environments unless intentionally sought. Third, the protocol depends on accurate localization of the BB, which limits the use of extremely small BBs. Fourth, validation of the fitting formulation in this proof‐of‐concept study was confined to SBP reconstructions. Although the modified Lorentzian parameterization was intentionally developed with sufficient structural flexibility to generalize beyond SBP, its performance under alternative reconstruction algorithms has not yet been systematically evaluated and remains a subject for future work. Fifth, the analytical simulation assumed a point focal spot, neglecting the finite three‐dimensional extent of a realistic X‐ray source. A spatially extended focal spot would broaden the effective PSF and may influence the uASF profile in ways not captured by the present model. Lastly, accurate estimation of FWHM required a reconstruction volume with finer inter‐slice spacing (0.2 mm) to enable reliable fitting. As many clinical systems reconstruct slices with 1 mm spacing, up‐sampling could introduce interpolation errors that were not explicitly examined here. This was not a concern in our experiments, as reconstructions were natively generated at high resolution.

With all this said the role of the z‐resolution test in routine quality control warrants further consideration. Under the current EUREF‐based framework, however, its utility may be limited. The 30% tolerance on FWHM stipulated by the American College of Radiology—perhaps to accommodate large variability inherent in the EUREF protocol revealed in this work—may be too broad to permit meaningful investigation of subtle failure modes.^18^ Performance deviations such as source‐position errors or reconstruction–geometry miscalibration could remain obscured within such a wide tolerance band. The heightened accuracy and precision of the Modified protocol, by contrast, would permit tighter tolerances while rendering the test more amenable to revealing such failure scenarios. Although integration into routine clinical workflows would require automation and system‐level support to ensure practical feasibility, the Modified protocol nevertheless remains particularly well suited for intercomparison between DBT systems and for the development of novel acquisition geometries. Because it tracks spatial variation in apparent angle with reduced sensitivity to noise, it is well positioned to monitor the stability of acquisition geometry over time and to isolate the effect of reconstruction algorithm changes independently of acquisition geometry or technique. These capabilities are especially relevant for NGT systems with patient‐specific source trajectories^19^ and for DBT units employing motion‐compensating focal spots,^20^, ^21^, ^22^ where geometry‐dependent variations in apparent angle, arising from over‐ or under‐compensation, would manifest as measurable changes in FWHM and could be sensitively detected by the Modified protocol. Future work will focus on extending the Modified protocol to clinical DBT reconstructions and assessing its compatibility across diverse imaging systems and acquisition geometries.

## 6 CONCLUSION

In conclusion, we demonstrate that the prevailing EUREF protocol for estimating z‐resolution in DBT is susceptible to systematic errors that limit its ability to faithfully reflect the underlying PSF. The proposed Modified protocol restores the expected linear relationship between FWHM and apparent angle, a surrogate for the local PSF structure, while substantially reducing stochastic bias in the reconstruction. The Modified protocol thus provides a geometry‐selective z‐resolution metric that may better support system intercomparison, acquisition geometry design, and more precise performance evaluation in DBT.

## CONFLICT OF INTEREST STATEMENT

Andrew D.A. Maidment and Raymond J. Acciavatti are inventors on patents and patent applications related to the next‐generation tomosynthesis (NGT) technology. Andrew D.A. Maidment is the spouse to an employee and shareholder of Real Time Tomography, LLC; is a member of the scientific advisory board of Real Time Tomography, LLC; and is an owner of Daimroc Imaging, LLC.

## Appendix

Here we theoretically derive the systematic errors within the EUREF protocol for anteriorly displaced BBs.

### Underestimation Factor

The FWHM reduction from PSF tilt stems from two effects: sampling along an intermediate axis instead of the true major axis, and projecting that profile onto the z‐axis. We formally quantify this reduction and its dependence on PSF orientation.

Referring to the 2D sagittal cross‐section of a tilted PSF (Figure 2a), we can compute the orientation of the intermediate sampling axis resulting from the EUREF protocol. The PSF is modeled as a tilted Gaussian (though not exact, this model is illustrative of general trends) defined over an orthogonal axis pair $\left(z^{\prime },r^{\prime }\right)$, where the major axis $z^{\prime }$ is oriented at an angle θ to the vertical z axis. In this view, a ROI line segment over which the maximum intensity is to be extracted, lies horizontally at some slice value say, $z=z_{i}$. The following expressions for the PSF's 2D cross‐section and the sampling ROI‐line may be written in the tilted $\left(z^{\prime },r^{\prime }\right)$ coordinate space,

(A1)
$$\mathrm{PS}F_{2D}\left(z^{\prime },r^{\prime }\right)=Ae^{-{\left(z^{\prime }/a\right)}^{2}}e^{-{\left(r^{\prime }/b\right)}^{2}}+C$$

(A2)
$$z^{\prime }=-\tan\theta r^{\prime }+z_{i}/\cos\theta$$

To locate the maxima of $\mathrm{PS}F_{2D}$ over this line, we employ the method of Lagrange multipliers with the Lagrangian,

(A3)
$$L\left(z^{\prime },r^{\prime }\right)\overset{\text{def}}{=}\mathrm{PS}F_{2D}\left(z^{\prime },r^{\prime }\right)+\lambda \left(z^{\prime }+\tan\theta r^{\prime }-z_{i}/\cos\theta \right)$$

This leads us to the coordinates of the tangency point

(A4)
$$\left(z_{*}^{\prime },r_{*}^{\prime }\right)=\frac{z_{i}/\cos\theta }{\left(\tan^{2}\theta +a^{2}/b^{2}\right)}\left(\frac{a^{2}}{b^{2}},\tan\theta \right)$$

It is evident that the locus of $\left(z_{*}^{\prime },r_{*}^{\prime }\right)$ for different $z_{i}$ slices, traces a linear path which makes an angle $\theta^{\prime }=\tan^{-1}\left(\frac{\tan\theta }{a^{2}/b^{2}}\right)$ relative to the PSF's major axis ($z^{\prime }$) and thus defines another axis called the **intermediate sampling axis (z″)**, which lies between the vertical axis (z) and the major axis ($z^{\prime }$). It is along ($z^{\prime \prime }$) that the ASF is effectively sampled due to the maxima‐based strategy of the EUREF protocol.

To compute the contraction in FWHM caused by this off‐axis sampling, we evaluate the ratio of two representative lengths— the distance from the origin (0,0) to the tangency point $\left(z_{*}^{\prime },r_{*}^{\prime }\right)$ that is R_eff_, and the distance from the origin to the ellipsoidal contour originating from $\left(z_{*}^{\prime },r_{*}^{\prime }\right)$ intersects the $z^{\prime }$‐major axis that is R_true_. Their ratio gives the sampling contraction as,

(A5)
$$\frac{R_{\mathrm{eff}}}{R_{\mathrm{true}}}=\frac{\sqrt{\tan^{2}\theta +a^{4}/b^{4}}}{\frac{a}{b}\sqrt{\tan^{2}\theta +a^{2}/b^{2}}}$$

Next, this sampled profile is then projected onto the vertical z‐axis introducing another contraction, equal to the cosine of the angle between the intermediate axis ($z^{\prime \prime }$) and the vertical axis (z) that is $\cos(\theta -\theta^{\prime })$. Thus, the **total underestimation in FWHM**—relative to the value that would have been measured along the true major axis of the PSF—is given by the product of the sampling and projection contraction ratios as,

(A6)
$$\begin{array}{cc} & \mathrm{Underestimation}\;\mathrm{Factor}\\ & =\frac{\sqrt{\tan^{2}\theta +a^{4}/b^{4}}}{\frac{a}{b}\sqrt{\tan^{2}\theta +a^{2}/b^{2}}}\cos\left(\theta -\tan^{-1}\left(\frac{\tan\theta }{a^{2}/b^{2}}\right)\right) \end{array}$$

### Overestimation Factor

Next the EUREF protocol computes the FWHM on this sampled profile. Ignoring the DC baseline in it results in an overestimation which we quantify as follows. We can consider the uASF profile generated from the 2D PSF to take the form $\mathrm{uASF}\left(z\right)=Ae^{-{\left(\frac{z}{a_{c}}\right)}^{2}}+C,$ accounting for the contraction derived earlier in $a_{c}$. Per the protocol, we normalize uASF(z) with respect to its peak value that is (A+C) and locate the FWHM as the width where it falls to 0.5. This gives us,

(A7)
$$\mathrm{FWH}M_{\mathrm{EUREF}}=2a_{c}\sqrt{\ln2}\sqrt{1+\frac{\ln\left(1+C/\left(A-C\right)\right)}{\ln2}}$$

Note, in the absence of a DC baseline (C=0), the $\mathrm{FWH}M_{\mathrm{EUREF}}$ would have matched the ground truth of $2a_{c}\sqrt{\ln2}$. However, because a SBP reconstruction will always contain a baseline amounting to the signal produced by 1 of the 15 projections (sans noise), this is not the case. The FWHM overestimation factor can be formally expressed as,

(A8)
$$\mathrm{Overestimation}\;\mathrm{Factor}=\sqrt{1+\frac{\ln\left(1+C/\left(A-C\right)\right)}{\ln2}}$$

### Cumulative Systematic Error Factor

We can now express the cumulative systematic error factor in the FWHM measured using the EUREF protocol as the product of the Underestimation and Overestimation factors.

(A9)
$$\begin{array}{cc} & \mathrm{Cumulative}\;\mathrm{Systematic}\;\mathrm{Error}\;\mathrm{Factor}\\ & =\frac{\sqrt{\tan^{2}\theta +a^{4}/b^{4}}}{\frac{a}{b}\sqrt{\tan^{2}\theta +a^{2}/b^{2}}}\cos\left(\theta -\tan^{-1}\left(\frac{\tan\theta }{a^{2}/b^{2}}\right)\right)\\ & \times \sqrt{1+\frac{\ln\left(1+\frac{C}{A-C}\right)}{\ln2}} \end{array}$$

We consider a representative case involving an elevated BB located above the breast support as height $\left(z_{0}\right)$. The PSF's obliquity angle (θ) as a function of anterior displacement (r) can be estimated to be $\theta =\tan^{-1}\frac{r}{\mathrm{SSD}-z_{0}}$ where SSD is the Source‐to‐Support distance. Assuming a 15‐view geometry and a noise‐less SBP reconstruction, we approximate the amplitude‐to‐baseline ratio of the BB's signal in the uASF(z) as A:C=14:1. Additionally, for a sharply elongated PSF typical of DBT, we set the ratio of the ellipsoidal axes to be a:b=10:1. For this scenario, we can plot the **Cumulative Systematic Error Factor** as a function of anterior displacement (Appendix Figure A1b).

### Analytical Simple Backprojection Formula

Under ideal conditions, the SBP reconstruction of a solid sphere admits an analytical expression. Consider a sphere of radius $r_{b}$ centered at $\vec{C},$ imaged by an X‐ray source at $\vec{S}$. To evaluate the SBP intensity at a point $\vec{R}$ in space we extend a ray from $\vec{S}$ toward $\vec{R}$. If this ray intersects the sphere, then the SBP value is the chord length of the ray within the sphere, otherwise it is zero. One can express the ray vector as $\vec{L}\left(\alpha \right)=\vec{S}+\alpha \left(\vec{R}-\vec{S}\right)$, where the scalar α∈[0,1] interpolates between $\vec{S}$ and $\vec{R}$. The chord length is obtained by solving for α at the intersection points, constrained by $∥\vec{L}\left(\alpha \right)-\vec{C}{∥}^{2}=r_{b}^{2}$. Expanding this yields a quadratic in α whose roots correspond to those two intersecting points.

(A10)
$$\alpha^{2}∥\vec{R}-\vec{S}{∥}^{2}+2\alpha \left(\vec{R}-\vec{S}\right)\cdot \left(\vec{S}-\vec{C}\right)+{∥\vec{S}-\vec{C}∥}^{2}-r_{b}^{2}=0$$

Solving A10 gives the analytical expression for the chord length,

(A11)
$$\mathrm{SBP}\left(\vec{R};\vec{S},\vec{C},r_{b}\right)=ℜ\sqrt{r_{b}^{2}-\frac{∥\left(\vec{R}-\vec{C}\right)\times {(\vec{S}-\vec{C})∥}^{2}}{∥\left(\vec{R}-\vec{S}\right){∥}^{2}}}$$

Here, ℜ is the real‐value operator. For all 15 X‐ray sources part of a DBT acquisition, we can average $\mathrm{SBP}(\vec{R};\vec{S},\vec{C},r_{b})$ in A11 to obtain noise‐less reconstructions of an ideal BB.

(A12)
$$\mathrm{SB}P_{\mathrm{recon}}=\frac{1}{15}\sum_{i=1}^{15}ℜ\sqrt{r_{b}^{2}-\frac{∥\left(\vec{R}-\vec{C}\right)\times {({\vec{S}}_{i}-\vec{C})∥}^{2}}{{∥(\vec{R}-{\vec{S}}_{i})∥}^{2}}}$$
